# HIV Infection Is Associated with Increased Fatty Infiltration of the Thigh Muscle with Aging Independent of Fat Distribution

**DOI:** 10.1371/journal.pone.0169184

**Published:** 2017-01-06

**Authors:** Javzandulam Natsag, Kristine M. Erlandson, Deborah E. Sellmeyer, Sabina A. Haberlen, Joseph Margolick, Lisa P. Jacobson, Frank J. Palella, Susan L. Koletar, Jordan E. Lake, Wendy S. Post, Todd T. Brown

**Affiliations:** 1 Johns Hopkins University, Baltimore, United States of America; 2 University of Colorado Anschutz Medical Campus, Aurora, United States of America; 3 Johns Hopkins Bloomberg School of Public Health, Baltimore, United States of America; 4 Northwestern University Feinberg School of Medicine, Chicago, United States of America; 5 The Ohio State University, Columbus, United States of America; 6 David Geffen School of Medicine at UCLA, Los Angeles, United States of America; Azienda Ospedaliera Universitaria di Perugia, ITALY

## Abstract

**Background:**

Lower muscle density on computed tomography (CT) provides a measure of fatty infiltration of muscle, an aspect of muscle quality that has been associated with metabolic abnormalities, weakness, decreased mobility, and increased fracture risk in older adults. We assessed the cross-sectional relationship between HIV serostatus, age, thigh muscle attenuation, and thigh muscle cross-sectional area (CSA).

**Methods:**

Mean CT-quantified Hounsfield units (HU) of the thigh muscle bundle and CSA were evaluated in 368 HIV-infected and 145 HIV-uninfected men enrolled in the Multicenter AIDS Cohort Study (MACS) Cardiovascular Substudy using multivariable linear regression. Models all were adjusted for HIV serostatus, age, race, and body mass index (BMI); each model was further adjusted for covariates that differed by HIV serostatus, including insulin resistance, hepatitis C, malignancy, smoking, alcohol use, and self-reported limitation in physical activity.

**Results:**

HIV-infected men had greater thigh muscle CSA (p<0.001) but lower muscle density (p<0.001) compared to HIV-uninfected men. Muscle density remained lower in HIV-infected men (p = 0.001) when abdominal visceral adiposity, and thigh subcutaneous adipose tissue area were substituted for BMI in a multivariable model. Muscle density decreased by 0.16 HU per year (p<0.001) of increasing age among the HIV-infected men, but not in the HIV-uninfected men (HIV x age interaction -0.20 HU; p = 0.002).

**Conclusion:**

HIV-infected men had lower thigh muscle density compared to HIV-uninfected men, and a more pronounced decline with increasing age, indicative of greater fatty infiltration. These findings suggest that lower muscle quality among HIV-infected persons may be a risk factor for impairments in physical function with aging.

## Introduction

Aging is associated with slow decline in muscle mass, quality, and function beginning in mid-life. For some individuals, this decline may result in impairments in physical function, disability, and frailty. Emerging data suggest that these complications may also develop at a relatively younger age in individuals with HIV infection, even among those with virologic suppression on effective antiretroviral therapy (ART). In a cross-sectional analysis of Multicenter AIDS Cohort Study (MACS) participants, HIV-infected men had a prevalence of the frailty phenotype greater than HIV-uninfected men, with the largest differences by HIV serostatus between ages 50 and 64 years [1]. The underlying etiology of frailty in HIV-infected populations is multifactorial, and likely includes effects of chronic HIV infection, effects of ART, an increased burden of chronic co-morbidities, and lifestyle and behavioral factors.

Abnormalities in fat distribution or lipodystrophy, including both central lipohypertrophy and peripheral lipoatrophy, are frequently described among HIV-infected individuals. Moreover, lipodystrophy has also been associated with abnormal fatty infiltration within skeletal muscle [2,3]. Therefore, one potential mechanism for functional decline in HIV-infected persons is lipid accumulation in and around skeletal muscle, or myosteatosis. Skeletal muscle contains intramyocellular lipid (IMCL) droplets within the cytoplasm of myocytes as well as intermuscular adipocytes. Abnormal increases in the skeletal muscle fat depot are thought to negatively affect muscle function by mechanical compression, and through secreted factors including hormones and cytokines [4,5]. Muscle density or attenuation, as measured in Hounsfield Units (HU) obtained by computed tomography (CT) scanning, is a noninvasive measure of muscle density that correlates with intramuscular lipid content assessment obtained by muscle biopsy [6,7]. Lower muscle density reflects higher intramuscular lipid content, which reflects a combination of IMCL and intermuscular adipocytes. Muscle density has been used to assess adiposity of the muscle and its functional correlates [8–12]. Lower CT-based skeletal muscle density is a predictor of decreased muscle function [7], and is associated with sarcopenia [13], increased fracture risk [8.9], mobility limitations [11], and frailty [14] in the general population.

We hypothesized that HIV infection would be associated with increased myosteatosis and decreased muscle mass. Using data from the MACS Cardiovascular Disease (CVD) Substudy [15], we investigated the association of HIV serostatus on muscle by examining thigh muscle density and cross-sectional area (CSA).

## Methods

### Study population

The MACS is an ongoing prospective cohort study of the natural and treated histories of HIV-1 infection in men who have sex with men, conducted in Baltimore, Maryland/Washington, DC; Chicago, Illinois; Pittsburgh, Pennsylvania; and Los Angeles, California [16]. The cohort includes both HIV-infected and HIV-uninfected men who attend semiannual research visits that include standardized interviews, physical examinations, and blood and urine collection for laboratory measurements [16].

Between 2010–2013, MACS men aged 40 to 70 years, weighing less than 300 pounds, and having no history of cardiac surgery or percutaneous coronary intervention were recruited into a CVD Substudy whose protocol included a mid-thigh CT scan [15]. Measures of thigh muscle density were completed in 607 men from the 2010–2013 CVD Substudy. Ninety-four participants had missing or invalid mid-thigh CT scans. The current analysis included 368 HIV-infected and 145 HIV-uninfected men. The institutional review boards (IRB) of each participating site (Johns Hopkins Bloomberg School of Public Health IRB and Johns Hopkins School of Medicine IRB [Baltimore], Western IRB [Washington, DC]; Northwestern University IRB [Chicago]; University of Pittsburgh Human Research Protection Office [Pittsburgh]; University of California Los Angeles Office of Human Research Protection Program and Harbor-University of California Los Angeles IRB [Los Angeles]) approved the study. All participants provided written, informed consent according to the approved IRB consent processes.

### Computed tomography and analysis procedures

CT images were analyzed by trained, experienced readers at the core CT reading center (Los Angeles Biomedical Research Institute at Harbor-UCLA) who were blinded to participant characteristics and HIV serostatus. Mid-thigh CT scans were obtained with a 3–10 mm cross-sectional single image for each subject at the midpoint between the anterior superior iliac crest and the patella. Manual tracings were performed to delineate thigh subcutaneous fat and muscle. Muscle density in HU was measured within the region bound by the fascia lata (outermost fascia) of the left thigh muscle. In image analysis, areas (cm^2^) of skeletal muscle and thigh subcutaneous adipose tissue (SAT) were measured by selecting the following regions of interest, defined by the following attenuation values described by Goodpaster et al [17]: -35 to -190 HU for adipose tissue, and 0–100 HU for muscle; and mean muscle attenuation was determined from all pixels within this range. CT was also used to measure abdominal visceral adipose tissue (VAT) and abdominal SAT areas (cm^2^) on a 3–10 mm cross-sectional single image obtained centered at the L4-L5 vertebral disc space. VAT area was quantified by delineating the intra-abdominal cavity at the internal-most aspect of the abdominal and oblique muscle walls and the posterior aspect of the vertebral body, excluding the psoas major. The body wall was manually traced, excluding all subcutaneous fat, using a spline tool. The internal region of interest was selected and area within the VAT range computed [18]. Abdominal SAT areas were calculated by subtracting intra-abdominal fat from total abdominal fat.

### Measurement of grip strength

Each semiannual MACS visit since 2006 has included an assessment of grip strength using a Jamar Dynamometer (Sammons Preston Rolyan, Bolingbrook, IL). The test was performed three times sequentially at the same MACS visit and an average was taken of the three measurements. We used the grip strength measurement closest in time to the CT scan visit within ± 4 visits (2 years). The median time between the CT scanning and the grip strength visits was 14 days, with an interquartile range (IQR) of -31 to 49 days.

### Biochemical and clinical parameters

Biochemical and clinical data were collected at the previous MACS visit closest to the CT scanning, generally within 6 months. Insulin concentrations were measured using radioimmunoassay (CV = 2.6%, Linco Research, St. Charles, MO). Fasting plasma glucose levels were measured by the combined hexokinase/glucose-6-phosphate dehydrogenase method (CV 1.8%). Glucose and insulin were processed at a central laboratory (Heinz Laboratory, Pittsburgh, PA). Insulin resistance was estimated using the Homeostasis Model Assessment of Insulin Resistance (HOMA-IR = [fasting plasma insulin (μU/ml) x fasting plasma glucose (mmol/ liter)]/ 22.5) at the substudy visit [19].

Alcohol consumption was categorized into none, mild to moderate (no more than 1–2 drinks per typical day for participants who consumed alcohol at least monthly, or no more than 3–4 drinks per typical day of drinking for participants who drank alcohol less than once a month), moderate to heavy (3–4 drinks per typical day for participants reporting alcohol on a monthly or more frequent basis, or ≥5 drinks per a day for participants who drank alcohol less than once a month), and binge drinking (≥5 drinks per a day at least once a month). Chronic hepatitis C virus (HCV) infection was defined as the presence of detectable HCV RNA. Diabetes was defined by self-report in addition to the use of diabetes medication or fasting glucose ≥ 126 mg/dL or hemoglobin A1c ≥ 6.5%. Self-reported limitations in physical activity were assessed using the SF-36 questionnaire; participants answered either “Yes, limited a lot”, “Yes, limited a little” or “No, not limited” to the question “Does your health now limit you in vigorous activities, such as running, lifting heavy objects, participating in strenuous sports?”. History of any malignancy was collected by self-report at each semi-annual visit and validated using clinical records. HIV-related variables included plasma HIV-1 RNA levels, CD4^+^ T lymphocyte counts, history of an AIDS-defining malignancy or opportunistic infection, and duration of highly active antiretroviral therapy (HAART) use (DHHS/Kaiser guidelines).

### Statistical methods

The distributions of demographic and clinical factors among HIV-infected and HIV-uninfected men were compared with muscle parameters using the Wilcoxon rank-sum test or chi square test, as appropriate. Univariate and multivariable linear regressions were performed using thigh-muscle density and CSA as outcome variables separately, with the covariates of HIV serostatus, age, race, and body mass index (BMI). Each model was further adjusted for covariates that differed by HIV serostatus (Table 1), which included HOMA-IR, history of any malignancy, cumulative pack-year smoking, degree of alcohol consumption, chronic HCV infection, and self-reported limitation in physical activity. When any of the muscle parameters were different by HIV serostatus, we added an HIV x age interaction term to explore if age modified the relationship between the muscle parameter and HIV serostatus. VAT and thigh SAT were compared by HIV serostatus using linear regression analysis adjusted for age, race and BMI. Further relationships were investigated between muscle parameters and VAT and thigh SAT, both of which were added to the multivariable model instead of BMI, and adjusted by age, race, HIV serostatus, HOMA-IR, history of malignancy, cumulative pack-year smoking, degree of alcohol consumption, chronic HCV infection, and self-reported limitations in physical activity. Correlation between grip strength and thigh muscle parameters was evaluated using Spearman's rank correlation.

**Table 1:** Study population characteristics.

Characteristics	HIV (-) n = 145	HIV (+)n = 368	P value
Age (year)	56.5 ± 6.4	53.8 ± 5.9	0.0001
Race			0.01
White	69.7%	58.7%	
Black	28.9%	34.8%	
Other	1.4%	6.5%	
BMI (kg/m^2^)	26.7 ± 4.0	26.2 ± 4.6	0.15
HOMA-IR (mmol/L x uU/mL)	3.6 ± 2	4.7 ± 4.3	0.03
Lipid lowering medication	35%	39%	0.4
GFR (ml/min/1.73m^2^)	90 ± 18	88 ± 24	0.2
History of malignancy	8%	14%	0.05
Diabetes Mellitus^†^	12%	19%	0.12
Chronic Hepatitis C Infection	6%	13%	0.02
Cumulative pack-year smoking	12 ± 18	15 ± 19	0.03
Alcohol consumption*			0.02
None	14%	24%	
Low to moderate	63%	60%	
Moderate to heavy	17%	11%	
Binge	6%	5%	
Use of opiate since last visit	2.2%	1.2%	0.4
Limitation in physical activity			0.4
None	54%	48%	
Limited a little	30%	33%	
Limited a lot	16%	19%	
CD4^+^ cell count (cells/mm3)^#^		607 (417; 799)	
Nadir CD4^+^ cell count (cells/mm3) ^#^		277 (156; 395)	
HIV-1 RNA <50 copies/ml (%)		83%	
Percent of individuals on HAART		89%	
Median duration of ART (years)^#^		9.7 (6.9; 12.5)	
Thigh muscle CSA (cm^2^)	140 ± 25	145 ± 26	0.09
Thigh muscle attenuation (HU)	52.1 ± 4	51.1 ± 3.9	0.002
Grip strength (kg)	35 ± 9	36 ± 9	0.46
VAT (cm^2^)	151 ± 89	169 ± 100	0.07
Abdominal SAT (cm^2^)	243 ± 113	203 ± 127	0.0001
Thigh SAT (cm^2^)	53 ± 27	33 ± 30	0.0001

Mean ± SD

^#^Median (Q1, Q3); BMI–Body mass index; HOMA-IR–homeostatic model assessment for insulin resistance; GFR–estimated glomerular filtration rate

^†^Diabetes Mellitus: self-reported DM plus the use of diabetes medication OR fasting glucose ≥ 126 mg/dL OR hemoglobin A1c ≥ 6.5%

*Alcohol consumption Low to moderate: 1–2 drinks/day or 3–4 drinks/day for no more than once a month, Moderate to heavy: 3–4 drinks/day for more than once a month or ≥5 drinks/day for less than once a month, Binge: ≥5 drinks/day for at least once a month; CSA–Cross sectional area; VAT–visceral adipose tissue; SAT–subcutaneous adipose tissue

Lastly, we explored factors associated with CSA and thigh muscle density in the HIV-infected men using linear regression modeling. In addition to demographic, metabolic, clinical, and behavioral factors, we examined associations with HIV-related factors (CD4^+^ T lymphocyte count, presence of detectable plasma HIV-1 RNA), and HIV treatment related variables (cumulative years use of any ART, protease inhibitor (PI), non-nucleoside reverse transcriptase inhibitor (NNRTI), zidovudine (AZT), stavudine (D4T), or tenofovir (TDF)). Variables that were associated with the outcome in univariate models were included in multivariable models. We also substituted measures of regional adiposity (VAT and thigh SAT) for BMI in the multivariable models. All statistical analyses were performed using Stata 13.1 (StataCorp, College Station, TX). Statistical significance was established at a two-sided p-value <0.05.

## Results

Among the 368 HIV-infected and 145 HIV-uninfected men, the HIV-infected men were younger, more likely to be black, and had higher HOMA-IR, greater cumulative pack year smoking history, were more likely to have chronic HCV infection, and were more likely to have a history of malignancy (Table 1). After adjustment for age, race and BMI, HIV-infected men had significantly greater VAT and significantly less thigh SAT, respectively (VAT difference = 41.7 cm^2^, 95% CI 27.2, 56.3; p<0.001; thigh SAT difference = -18.2 cm^2^, 95% CI -22.8, -13.6; p<0.001) compared to HIV-uninfected men.

### Thigh muscle cross-sectional area

As shown in Table 2, greater thigh muscle CSA was correlated with lower age and increased BMI, VAT, thigh SAT, and grip strength. In models adjusted for age, race, and BMI, HIV-infected men had greater thigh CSA (difference = 7.1 cm^2^; 95% CI 2.8, 11.4; p<0.001) compared to HIV-uninfected men; these associations remained significant when the model was further adjusted for HOMA-IR, malignancy, cumulative pack-year smoking, alcohol use, chronic HCV infection, and self-reported limitation in physical activity (Table 3). There was no interaction between age and HIV (p = 0.58).

**Table 2:** Pearson Correlations (r) between age, BMI, grip strength, thigh muscle cross sectional area, thigh muscle density (TMD), visceral adipose area, thigh subcutaneous adipose area and insulin resistance among HIV-infected and HIV-uninfected men.

	Age	BMI	Grip	Thigh CSA	TMD	VAT	Thigh SAT
BMI	-0.08						
Grip	-0.03	0.04					
Thigh CSA	-0.24*	0.55*	0.26*				
TMD	-0.11^#^	-0.25*	0.09^#^	0.12*			
VAT	0.21*	0.55*	-0.03	0.17*	-0.29*		
Thigh SAT	-0.08	0.58*	-0.07	0.13*	-0.2*	0.09^#^	
HOMA-IR	-0.01	0.3*	-0.1^#^	0.15^$^	-0.09	0.32*	0.05

CSA = cross sectional area; TMD = thigh muscle density (HU); VAT = visceral adipose tissue; SAT = subcutaneous adipose tissue; HOMA-IR = homeostasis model assessment for insulin resistance

* P<0.001

^$^P<0.01

^#^P<0.05

**Table 3:** Differences in Muscle CSA and attenuation between HIV-infected vs uninfected groups.

Covariate	Model 1		Model 2	
	Beta coefficient difference	P value	Beta coefficient difference	P value
Thigh muscle CSA (cm2)	7.1 (CI 2.8; 11.4)	<0.001	10.2 (CI 5.7; 14.7)	<0.001
Thigh muscle attenuation (HU)	-1.6 (CI -2.4; -0.9)	<0.001	-1.5 (CI -2.3; -0.7)	<0.001

Model 1 –adjusted with age, race, BMI; Model 2 –Adjusted for age, race, BMI, HOMA-IR, malignancy, cumulative pack-year smoking, alcohol consumption, chronic HCV infection and physical limitation; CSA–Cross sectional area; HU–CT scan Hounsfield Unit

We next explored the effect of HIV-serostatus on thigh CSA, when VAT and thigh SAT were substituted for BMI in the multivariable model. The magnitude of difference in thigh muscle CSA by HIV serostatus was attenuated and statistical significance was lost (difference = 3.6 cm^2^; 95% CI -2.0, 9.2; p = 0.21). In this model, VAT was strongly associated with higher thigh CSA (9.9 cm^2^ per ln increase (95% CI: 6.2, 13.7; p<0.001), but thigh SAT was not (1.1 cm^2^ per ln increase (95% CI: -1.5, 3.8, p = 0.41).

In univariate models restricted to HIV-infected men (S1 Table), lower thigh muscle cross-sectional area was associated with older age, white (vs black) race, lower BMI, lower VAT, lower thigh SAT, lower HOMA-IR, chronic HCV infection, cumulative smoking, binge alcohol drinking, and lower current CD4 cell count. In the multivariable model including BMI (Model A, S1 Table), lower CSA was associated with older age, lower BMI, and binge alcohol drinking. Similar results were obtained when VAT and thigh SAT were substituted for BMI (Model B), although race (black vs white race, 10.2 cm^2^ (3.2, 17.2)) and chronic hepatitis C infection (-9.7 cm^2^ (-18.4, -1.04)) became statistically significant. To help explain the difference in the effect of race between Models A and B, we compared BMI and VAT by race. Interestingly, whereas BMI was similar in the black and white men (median 25.7 vs 25.4 kg/m^2^, p = 0.66), VAT was lower in the black men then the white men (ln VAT 4.6 vs 5.2 cm^2^, p<0.001).

### Thigh muscle density

**Fig 1:**
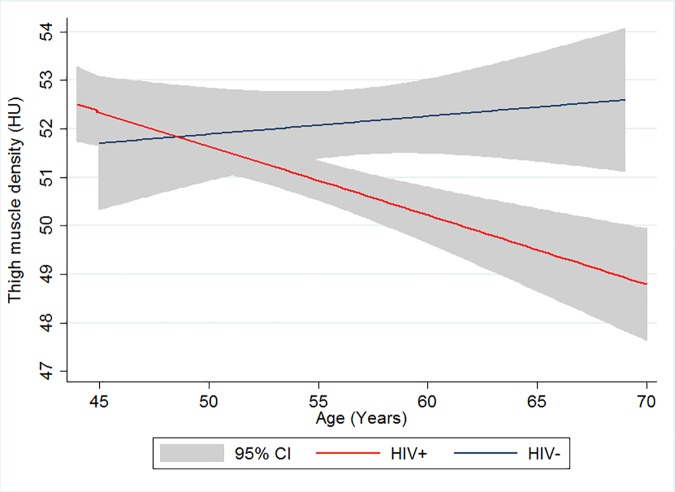
Association of thigh muscle density and age among HIV-infected (red) and HIV-uninfected (blue) men in the MACS Cohort.

Lower thigh muscle density (fattier muscle) was correlated with greater VAT, thigh SAT, BMI, and weaker grip strength (Table 2). In models adjusted for age, race, and BMI, HIV-infected men had lower thigh muscle density (difference = -1.6 HU; 95% CI -2.4, -0.9; p<0.001) compared to HIV-uninfected men (Table 3). Results were similar after adjustment for additional variables which differed by HIV-serostatus (Table 3, Model 2). Thigh muscle density decreased by 0.16 HU per year (95% CI -0.23, -0.08; p<0.001) with increasing age among the HIV-infected men, but did not change significantly with age in the HIV-uninfected men (HIV x age interaction -0.20 HU; 95% CI -0.32; -0.07; p = 0.002) (Fig 1).

Thigh muscle density remained lower in HIV-infected men (difference = -1.6 HU; 95% CI -2.4, -0.76; p<0.001) even when VAT and thigh SAT were substituted for BMI in the multivariate model, suggesting that HIV-infection is associated with lower muscle density independent of fat distribution.

In univariate models restricted to the HIV-infected men, lower thigh muscle density was associated with older age, higher BMI, higher VAT, higher thigh SAT, cumulative smoking and binge alcohol drinking (Table 4) while cumulative D4T and TDF use were associated with higher thigh muscle density. In the multivariable model (Table 4) including BMI, lower thigh muscle density was associated with older age, higher BMI, cumulative smoking, and binge alcohol drinking, and less cumulative D4T use. Similar results were obtained when VAT and thigh SAT were substituted for BMI (data not shown).

**Table 4:** Factors Associated with Thigh Muscle Density (TMD) among HIV-infected Men. Multivariable model includes age, race, BMI plus other factors associated with TMD in univariate models.

	Univariate	Multivariable
Age (per year)	-0.14 (-0.21,-0.08)*	-0.20 (-0.27, -0.13)*
Race (vs White (ref))		
Black	-0.16 (-1.0, 0.69)	-0.47 (-1.3, 0.36)
Other	0.97 (-0.68, 2.6)	-0.28 (-1.9, 1.4)
BMI (per kg/m^2^)	-0.24 (-0.33, -0.15)*	-0.26 (-0.34, 0.17)*
Metabolic/Clinical Factors		
ln HOMA-IR	-0.33 (-0.98, 0.32)	
VAT (per ln cm^2^)	-1.3 (-1.6, -0.79)*	
Thigh SAT (per ln cm^2^)	-1.2 (1.3, 6.6)*	
History of Malignancy	-0.72 (-1.9, 0.44)	
Estimated GFR (per ml/min/1.73m^2^)	-0.001 (-0.02, 0.009)	
Chronic HCV Infection	-0.13 (-1.2,1.2)	
Behavioral Factors		
Cumulative smoking (per pack-year)	-0.04 (-0.29, -0.01)*	-0.02 (-0.05, -0.004)*
Alcohol consumption (vs none)		
Low to moderate	-0.7 (-0.23, 1.7)	0.27(-0.63, 1.2)
Moderate to heavy	0.433 (-1.1, 1.7)	0.04 (-1.4, 1.5)
Binge	-2.5 (-4.5, -0.53)*	-3.0 (-4.9, -1.1)*
Use of opiate since last visit	-1.0 (-4.8, 2.8)	
HIV-Related Factors		
CD4^+^ cell count (per cell/mm3)	0.0001 (-0.001, 0.001)	
Nadir CD4 cell count (per cell/mm3)	0.001 (-0.001, 0.003)	
HIV-1 RNA (<50 copies/ml ref)	-0.21 (-1.3, 0.86)	
ART Factors		
Cumulative HAART use (per year)	0.06 (-0.04, 0.15)	
Cumulative PI (per year)	0.31 (-0.04, 0.10)	
Cumulative AZT (per year)	-0.03 (-0.12, 0.06)	
Cumulative d4T (per year)	0.23 (0.08, 0.37)*	0.21 (0.07, 0.35)*
Cumulative TDF (per year)	0.17 (0.03, 0.31)*	0.13 (-0.01, 0.26)

*P<0.05

BMI–Body mass index;HOMA-IR–homeostatic model assessment for insulin resistance; VAT–visceral adipose tissue; SAT–subcutaneous adipose tissue; GFR–estimated glomerular filtration rate

## Discussion

In this well-characterized cohort of HIV-infected and similar HIV-uninfected men, we found that HIV-infected men had greater thigh muscle mass but lower thigh muscle density, indicative of greater of fatty skeletal muscle infiltration. In addition, both lower thigh muscle CSA and density correlated with weaker grip strength. Furthermore, lower thigh muscle density was associated with advancing age among HIV-infected men only. Taken together, our findings suggest that fatty infiltration of the muscle, often associated with aging, is more pronounced in HIV-infected men. These findings may have relevant implications for the development of physical function impairment, frailty and fractures, in middle-aged and older HIV-infected populations.

To our knowledge, this is the largest study to compare muscle density by HIV serostatus. Previous studies examining fatty infiltration of the muscle in HIV-infected persons have focused on those with HIV-associated lipodystrophy [2,3,20]. Torriani et al [2] found that psoas muscle density on CT was significantly lower in HIV-infected men with lipodystrophy, with median density of 55 HU compared with HIV-infected men without lipodystrophy (57 HU) and HIV-uninfected controls (59.5 HU). The median psoas muscle density was higher than observed in our study population, which may be due to the nearly 10-year difference in age between the two study populations. Torriani et al similarly found that higher VAT was significant predictor of lower psoas muscle density. However, we further found that thigh muscle density remained significantly lower among HIV-infected men, independent of VAT and thigh SAT areas. Although we were unable to detect a relationship between muscle density and insulin resistance, prior studies measuring muscle fatty infiltration using ^1^H-magnetic resonance spectroscopy have found an association between increased intramyocellular fat and insulin resistance among HIV-infected lipodystrophic individuals [2, 20, 21].

In the general population, muscle density declines with advancing age [7,13,22,23]. The cross-sectional association with age and lower muscle density in HIV-infected but not uninfected men in our study may suggest a faster rate of age-related decline in muscle density (i.e. increase in fatty infiltration) among HIV-infected persons. A prior study by Taafe, et al, reported a 4–5 HU loss followed by 2–3 HU gain in muscle density with detraining and retraining among older, HIV-uninfected adults, thus our small (-1.5 HU) differences by HIV serostatus are likely clinically meaningful [24]. Furthermore, the interaction by age and HIV serostatus suggests that magnitude of the difference by HIV serostatus may increase at older ages. We speculate that increased muscle fat content reflects early decline in muscle quality, preceding the loss of lean muscle mass and decline in muscle performance and physical function.

D4T, an early thymidine analogue NRTIs, is strongly associated with lipoatrophy, mitochondrial toxicity, and adipocyte apoptosis [25]. Given the known relationship between D4T use and hepatic steatosis [26], we had hypothesized that D4T use would be associated with a greater degree of fatty infiltration of the muscle. In contrast to our hypothesis, we found the opposite: cumulative D4T use was associated with higher thigh muscle density (i.e., less fatty infiltration). These findings suggest that the effect of D4T on muscle fat could be more similar to fat depletion changes in subcutaneous fat or bone marrow fat [27]. ART treatment regimens are heterogeneous and vary over time within individuals, thus limited conclusions can be made regarding ART effects in the context of cross-sectional studies. Furthermore, participants with prior D4T exposure had longer exposure to all ART, and only four participants were receiving D4T at the time of CT assessment. Thus our results may simply represent a chance finding.

Contrary to our hypothesis, HIV-infected men had greater thigh muscle CSA compared to HIV-uninfected men, after adjustment for age, race and BMI, but not when adjusted for VAT and thigh SAT. The HIV-infected men tended to have higher VAT than the HIV-uninfected men, and VAT, but not thigh SAT, was strongly related to CSA. Taken together, these results suggest that apparent differences by HIV serostatus in thigh CSA in the univariate and BMI-adjusted models may be related to differences in fat distribution, particularly increased VAT. Prior studies such as the FRAM study [28], have found lower total skeletal muscle mass measured by magnetic resonance imaging among HIV-infected men compared to controls, possibly due to increased HIV disease severity: in the FRAM study, 40% of the HIV-infected group had detectable HIV-1 RNA, and the median CD4^+^ T lymphocyte count (391 cell/μL) was lower than in our cohort. Furthermore, BMI was significantly lower in the FRAM HIV-infected group compared to the controls, and muscle mass was not adjusted for BMI or fat distribution [28].

Our study had several limitations. First, the MACS population includes only men and our findings may not be generalizable to women. It is possible that the presence of hormonal abnormalities, such as hypogonadism, may be important in the relationship between HIV and thigh muscle density; the role of hormonal alterations in muscle fat infiltration should be further investigated in future analyses. Our sample was over 30% non-white. While we adjusted all analyses for race, there are likely complex relationships between race, body composition, and muscle parameters. For example, the effect of race on CSA was only apparent after the effects of VAT were taken into account. Given that VAT was lower in the black men than the white men, despite similar BMI, the differential effect of race on CSA when BMI or regional fat was included in the multivariable model (S1 Table) most likely represents negative confounding. These relationships should be explored in further studies. Next, although lower thigh muscle density was associated with lower grip strength, the relationship between muscle density and strength may have been much more apparent if we were able to test thigh muscle strength. Unfortunately, this measurement was not available. Furthermore, we did not have objective measures of physical activity or dietary assessments. Many HIV-infected men in the MACS have a history of significant immunosuppression and are highly ART experienced. Although we have attempted to examine these factors in the analysis, it is possible that the age effects with respect to muscle parameters that we observed in this analysis among HIV-infected men are a reflection of longer exposure to toxic ART and previous immunosuppression. Finally, the cross-sectional design limits the causal inferences that we can draw from the observed associations.

In summary, HIV-infected men have lower CT-quantified thigh muscle density, a measure of fatty infiltration and indicative of lower muscle quality, compared to HIV-uninfected men. In addition, we found that HIV-infected men had a more pronounced decline in thigh muscle density with increasing age. These findings suggest impairments in muscle quality among HIV-infected persons that may have implications for functional decline, impaired mobility, falls, and fracture risk with aging. Longitudinal research is required to confirm our finding in additional populations, and future studies should seek to identify interventions to prevent or reverse HIV-associated myosteatosis and subsequently limit physical function decline among aging HIV-infected persons.

## Supporting Information

S1 Table Factors Associated with Thigh Cross Sectional Area (CSA) Among HIV-infected Men. Model A includes BMI plus other factors associated with CSA in univariate models. Model B includes the same variables as Model A, but regional fat measures are substituted for BMI.
(DOCX)
