# Supplementary material for: HIV Infection Is Associated with Increased Fatty Infiltration of the Thigh Muscle with Aging Independent of Fat Distribution
Source: PLoS One. 2017 Jan 6;12(1):e0169184. doi: 10.1371/journal.pone.0169184 (PMC5218482; doi:10.1371/journal.pone.0169184)
Supplement: S1 Table — Model A includes BMI plus other factors associated with CSA in univariate models. Model B includes the same variables as Model A, but regional fat measures are substituted for BMI. (DOCX) [file pone.0169184.s001.docx]

**Supplemental Table 1**. Factors Associated with Thigh Cross Sectional Area (CSA) Among HIV-infected Men. Model A includes BMI plus other factors associated with CSA in univariate models. Model B includes the same variables as Model A, but regional fat measures are substituted for BMI.

|  | Univariate | Model A | Model B |
| --- | --- | --- | --- |
| Age ( per year) | -1.1 (-1.5,-0.6)* | -0.61 (-1.0, -0.20)* | -1.1 (-1.6, -0.66)* |
| Race (vs White (ref)) |  |  |  |
| Black | 5.7 (0.3, 11.5)* | 3.6 (-1.6, 8.9) | 10.2 (3.2, 17.2)* |
| Other | 5.2 (-5.8, 16.3) | -5.9 (-16.1, 4.2) | -3.4 (-14.7, 8.0) |
| BMI ( per kg/m^2^) | 3.4 (2.9, 3.9)* | 3.2 (2.7, 3.7)* | - |
| Metabolic/Clinical Factors | | | |
| ln HOMA-IR | 8.1 (3.8, 12.3)* | -0.14 (-4.1, 3.9) | 4.4 (-0.28, 9.1) |
| VAT (per ln cm^2^) | 9.0 (5.3, 12.7)* | - | 10.8 (6.1,15.5)* |
| Thigh SAT (per ln cm^2^) | 3.9 (1.3, 6.6)* | - | 1.3 (-1.6, 4.2) |
| History of Malignacy | -7.1 (-14.9, 0.64) |  |  |
| Estimated GFR (per ml/min/1.73m^2^) | 0.6 (-0.04, 0.18) |  |  |
| Chronic Hepatitis C infection | -9.6 (-17.7, -1.52)* | -6.6 (-14.1, 0.98) | -9.7 (-18.4, -1.04)* |
| Behavioral Factors | | | |
| Cumulative smoking (per pack-year) | -0.15 (-0.29, -0.01)* | -0.0.10 (-0.22, 0.03) | -0.10 (-0.23, 0.04) |
| Alcohol consumption (vs none) |  |  |  |
| - Low to moderate | -1.0 (-7.6, 5.5) | 0.36(-5.1, 5.8) | -0.48 (-6. 7, 5.7) |
| - Moderate to heavy | 0.48 (-9.5, 10.5) | 3.1 (-5.6, 11.8) | 0.03 (-9.5, 9.5) |
| Binge | -14.2 (-27.9, -0.44)* | -12.0 (-23.3, -0.67)* | -11.6 (-24.3, 1.0) |
| Use of opiate since last visit | -15.6 (-40.8, 9.7) |  |  |
| HIV-Related Factors | | | |
| CD4^+^ cell count (per cell/mm3) | 0.01 (0.002, 0.02)* | 0.008 (-0.002, 0.14) | 0.007 (-0.002, 0.02) |
| Nadir CD4 cell count (per cell/mm3) | 0.01 (-0.001, 0.03) |  |  |
| HIV-1 RNA (<50 copies/ml ref) | -3.1 (-10.3, 4.2) |  |  |
| ART Factors | | | |
| Cumulative HAART use (per year) | 0.03 (-0.63, 0.69) |  |  |
| Cumulative PI (per year) | 0.12 (-0.37, 0.60) |  |  |
| Cumulative AZT (per year) | 0.32 (-0.30, 0.95) |  |  |
| Cumulative d4T (per year) | 0.005 (-0.96, 0.97) |  |  |
|  |  |  |  |

*P<0.05; BMI – Body mass index; HOMA-IR – homeostatic model assessment for insulin resistance; VAT – visceral adipose tissue; SAT – subcutaneous adipose tissue; GFR – estimated glomerular filtration rate
